# Supplementary material for: USP19 deubiquitinase inactivation regulates α-synuclein ubiquitination and inhibits accumulation of Lewy body-like aggregates in mice
Source: NPJ Parkinsons Dis. 2023 Nov 28;9:157. doi: 10.1038/s41531-023-00601-1 (PMC10684503; doi:10.1038/s41531-023-00601-1)

Supplementary Table 1 – List of Antibodies and Oligonucleotides

REAGENT	SOURCE	IDENTIFIER
**Antibodies**		
Mouse anti-a-Synuclein (Syn-1)	BD Bioscience	610787
Mouse anti-alpha-synuclein (4D6)	Abcam	ab1903
Mouse anti-ubiquitin (FK2)	Millipore	ST1200
Rabbit anti-ubiquitin	Sigma	U5379
Rabbit anti-pS129-a-Synuclein (EP1536Y)	Abcam	ab51253
Mouse anti-pS129-a-Synuclein (81A)	Abcam	ab184674
Rabbit anti-phospho-a-Synuclein (Ser129) (D1R1R)	Cell Signaling	23706
Guinea pig anti-MAP2	Synaptic Systems	188004
Rabbit anti-Tyrosine Hydroxylase (TH)	Millipore	AB152
Rabbit anti-NeuN	Abcam	ab177487
Rabbit anti-NeuN	Millipore	MAB377
Mouse anti-Iba-1	Wako	019-19741
Rabbit anti-Iba-1	Wako	016-20001
Rabbit anti-GFAP	Abcam	ab7260
Mouse anti-GFAP	Santa Cruz	sc-33673
Mouse anti-GFAP	Millipore	MAB360
Mouse anti-pTau (Ser202, Thr205) AT8	Thermo Scientific	MN1020
Mouse anti-Tau (TAU-5)	Thermo Scientific	AHB0042
Rabbit anti-USP19	Abcam	ab167059
Mouse anti-SNAP25	Synaptic Systems	111011
Mouse anti-Synaptotagmin-1	Synaptic Systems	105011
Alexa 488 goat anti-mouse	Invitrogen	A11029
Alexa 568 goat anti-mouse	Invitrogen	A11004
Alexa 647 donkey anti-mouse	Invitrogen	A31571
Alexa 488 goat anti-rabbit	Invitrogen	A11008
Alexa 568 goat anti-rabbit	Invitrogen	A11011
Alexa 647 donkey anti-rabbit	Invitrogen	A31573
Alexa 555 goat anti-guinea pig	Invitrogen	A21435
Opal 520	Akoya Biosciences	SKU FP1487001KT
Opal 570	Akoya Biosciences	SKU FP1488001KT
Opal 690	Akoya Biosciences	SKU FP1497001KT
HRP-conjugated anti-mouse	Biorad	1705047
HRP-conjugated anti-rabbit	Biorad	1705046
**Oligonucleotides**		
RNAscope USP19-common probe	ACDbio	Mm-Usp19
RNAscope USP19-ER probe	ACDbio	Mm-Usp19-O1-C2
RNAscope USP19-cyt probe	ACDbio	Mm-Usp19-O2-C3
Geo cassette Forward	IDT	CAGCAACCAGTAACCTCTG
Geo cassette Reverse	IDT	GACAGTATCGGCCTCAGGAAGATCG
USP19 Forward	IDT	GTAAAGATGGAGATGCTAGGAG
USP19 Reverse	IDT	GCTTAACAATCACCTCATCTGC

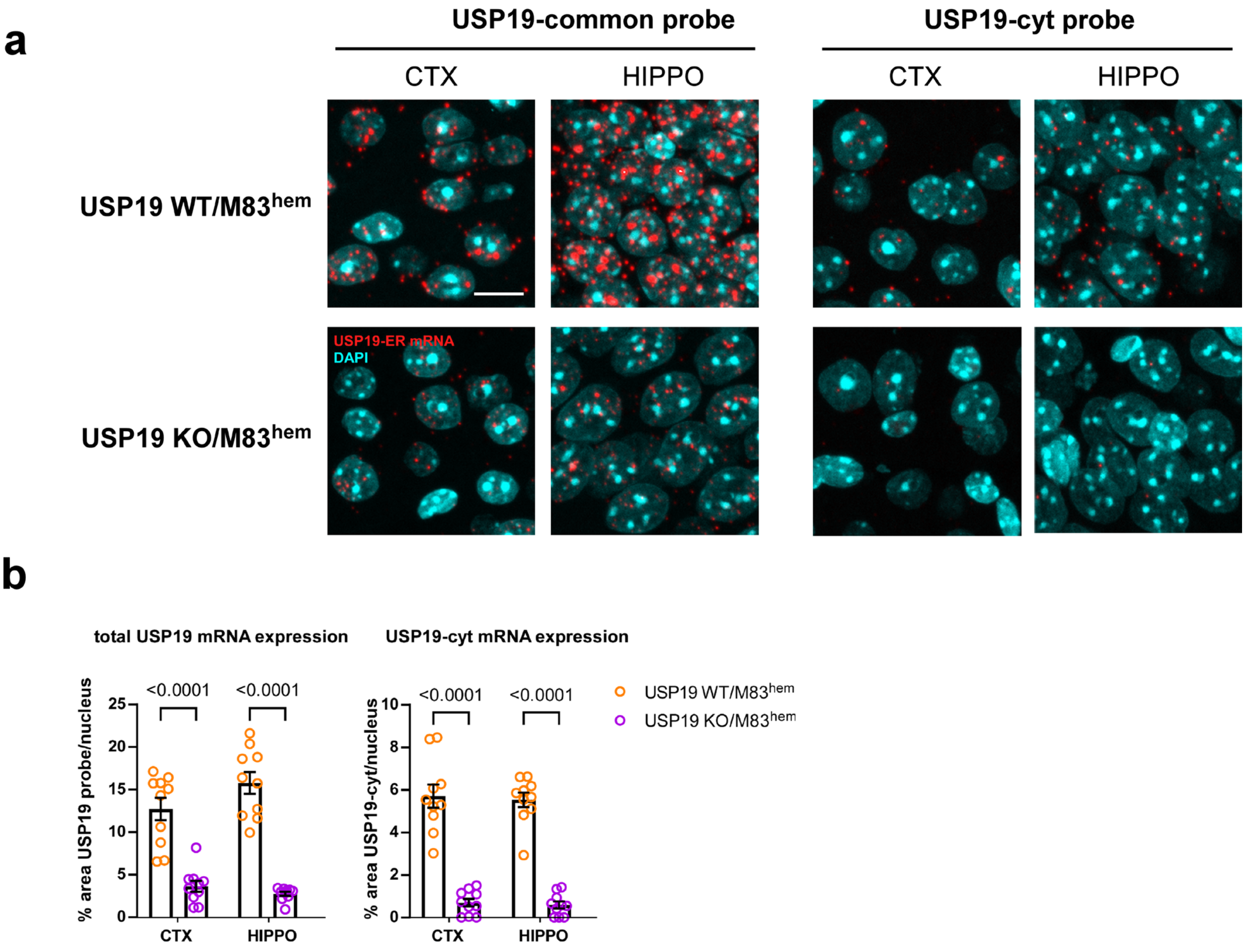

Supplementary Fig. 1: USP19 cytoplasmic isoform is expressed in the brain. a. RNAScope in-situ hybridization on FFPE brain sections to detect the presence (red signal) of both ER and cytoplasmic USP19 (using common probes) and cytoplasmic only USP19 isoform in USP19 WT/M83hem and USP19 KO/M83hem animals. Shown are representative images of CTX and HIPPO. DAPI was used to label nuclei. Scale bar= 10µm. **b.** Quantification of RNAScope USP19-common and -cyt signals presented as percent area of red pixels in 10 randomly selected nuclei in CTX and HIPPO. Data are mean ± s.e.m. Unpaired t-test was used for statistical analysis. P-values are indicated.

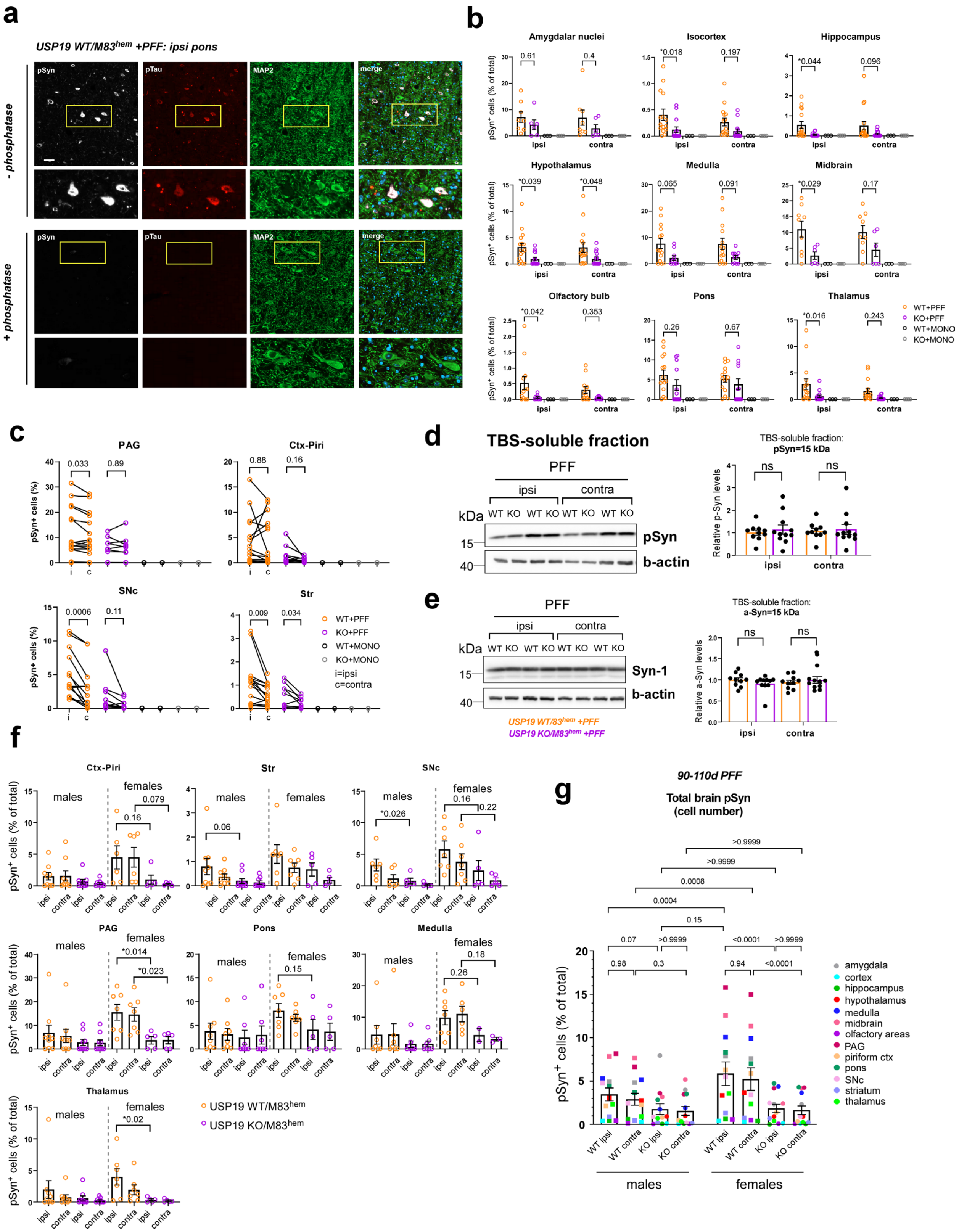
a
USP19 WT/M83hem +PFF: ipsi pons
- phosphatase
+ phosphatase
pSyn
pTau
MAP2
merge
b
Amygdalar nuclei
Isocortex
Hippocampus
Hypothalamus
Medulla
Midbrain
Olfactory bulb
Pons
Thalamus
pSyn+ cells (% of total)
ipsi
contra
0.61
0.4
*0.018
0.197
*0.044
0.096
*0.039
*0.048
0.065
0.091
*0.029
0.17
*0.042
0.353
0.26
0.67
*0.016
0.243
WT+PFF
KO+PFF
WT+MONO
KO+MONO
c
PAG
Ctx-Piri
SNc
Str
pSyn+ cells (%)
0.033
0.89
0.88
0.16
0.0006
0.11
0.009
0.034
i=ipsi
c=contra
d
TBS-soluble fraction
PFF
kDa
WT KO WT KO WT KO WT KO
pSyn
b-actin
TBS-soluble fraction:
pSyn=15 kDa
Relative p-Syn levels
ns
e
Syn-1
TBS-soluble fraction:
a-Syn=15 kDa
Relative a-Syn levels
USP19 WT/83hem +PFF
USP19 KO/M83hem +PFF
f
Ctx-Piri
Str
SNc
PAG
Pons
Medulla
Thalamus
males
females
pSyn+ cells (% of total)
0.079
0.16
0.06
*0.026
0.16
0.22
*0.014
*0.023
0.15
0.18
0.26
*0.02
USP19 WT/M83hem
USP19 KO/M83hem
g
90-110d PFF
Total brain pSyn
(cell number)
>0.9999
>0.9999
0.0008
0.0004
0.15
0.07
>0.9999
<0.0001
>0.9999
0.98
0.3
0.94
<0.0001
pSyn+ cells (% of total)
WT ipsi
WT contra
KO ipsi
KO contra
males
females
amygdala
cortex
hippocampus
hypothalamus
medulla
midbrain
olfactory areas
PAG
piriform ctx
pons
SNc
striatum
thalamus

Supplementary Fig. 2: Levels of total soluble pSyn and total soluble α-syn in USP19-WT and -KO PD-like brains. a. Immunofluorescence images of pSyn (A) and pTau (B), and neuronal (MAP2) and DAPI staining of brain sections (shown is ipsi pons) treated or not with phosphatase to test the specificity of pSyn and pTau staining. Scale bar= 50µm. **b.** Quantification of pS129-Syn$^+$ cells using QuPath analysis in indicated brain regions. Each data point represents a mean of 4 brain sections per animal. n ≥ 10 (PFF-injected) and n=3-4 (α-syn monomers-injected) biologically independent animals. Data are mean ± s.e.m. Two-way ANOVA was used for statistical analysis followed by Sidak multiple comparisons test. **c.** Paired analysis of pS129-Syn$^+$ cells in indicated brain regions showing ipsi- to contralateral region spreading of pSyn pathology. Each data point represents a mean of 4 brain sections per animal. n ≥ 10 (PFF-injected) and n=3-4 (α-syn monomers-injected) biologically independent animals. Data are mean ± s.e.m. Paired t-test was used for statistical analysis. **d.-e.** Representative immunoblots of pS129-Syn and total α-syn (Syn-1) of TBS-soluble whole hemisphere fractions. Beta-actin was used as a loading control. Data are mean ±s.e.m., n ≥ 10 biologically independent animals. **f.** Quantification of pS129-Syn$^+$ cells using QuPath analysis in indicated brain regions in male and female mice. Each data point represents a mean of 4 brain sections per animal. n ≥ 4 PFF-injected biologically independent male or female mice, except for medulla region (n≥2). Data are mean ± s.e.m. Two-way ANOVA was used for statistical analyses followed by Sidak multiple comparisons test. P-values are indicated. **g.** Overall quantification of total brain pS129-Syn represented as pSyn$^+$ cells (% of total) using IHC in A with additional brain regions at 90-110 dpi. Each point is a mean of means per brain region (each brain region is color-coded). Data are mean ±s.e.m. Two-way ANOVA was used for statistical analysis followed by Tukey's multiple comparisons test. P-values are indicated.

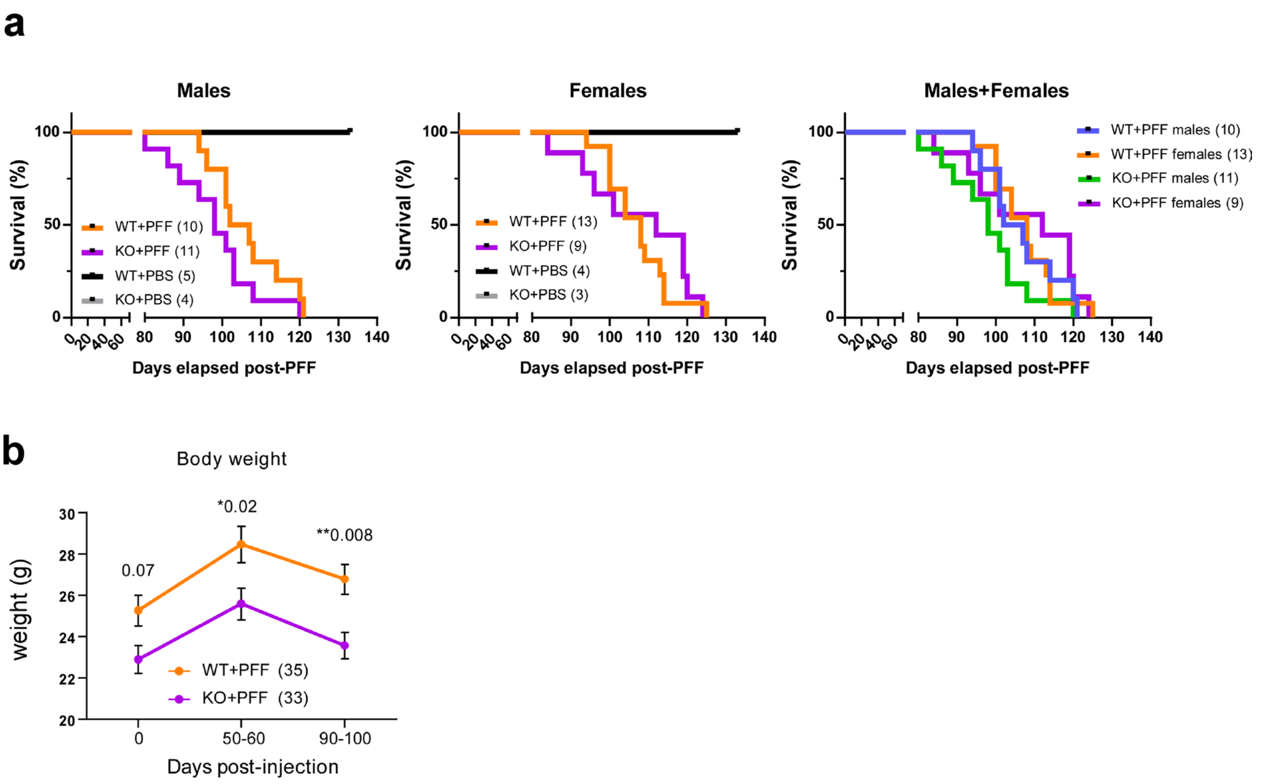

Supplementary Fig. 3: Survival of USP19 WT and KO PD-like mice

a. Kaplan-Meier survival analysis of USP19 WT/M83^{hem} and KO/M83^{hem} males and females injected with PFF or PBS. n≥ 3 biologically independent animals. Statistical analysis for survival curves was performed by long-rank (Mantel-Cox) test. **b**. Effects of USP19 depletion on body weight in PD-like mice. KO animals have significantly reduced body weight compared to WT which may lead to a faster deterioration upon the manifestation of PD-like symptoms. Data are mean ±s.e.m. Two-way ANOVA was used for statistical analysis followed by Sidak's multiple comparisons test. P-values are indicated.

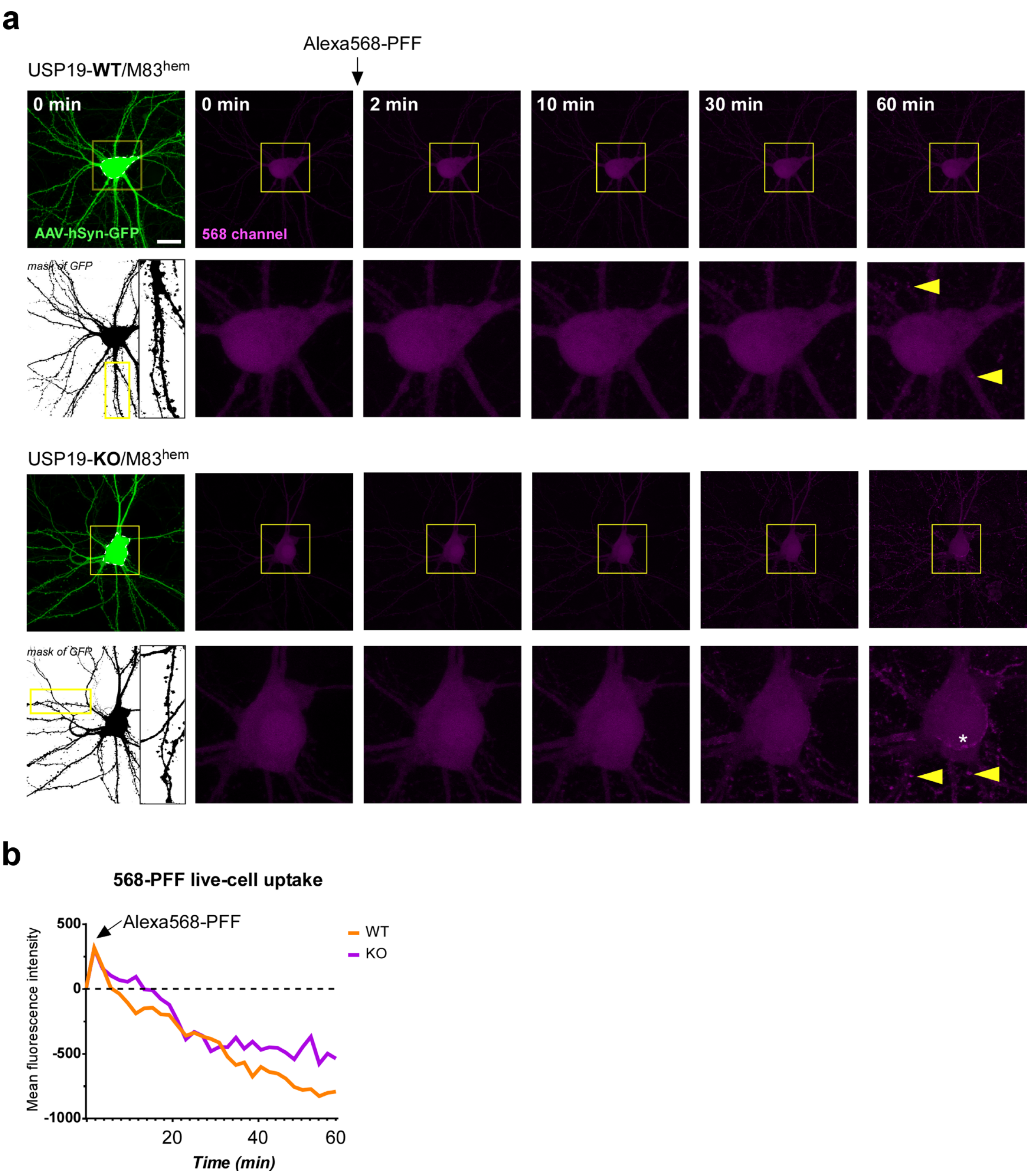

Supplementary Fig. 4: Live-cell PFF uptake in USP19 WT and KO neurons.

a. Micrographs of live-cell imaging of Alexa568-PFF (magenta) uptake in WT and KO neurons. GFP-expressing mature (20 DiV) spiny neurons (B/W mask inserted for better visualization) were treated with 1 μg/ml Alexa568-PFF and mean fluorescence intensity was measured in the cell body as measure of

uptake over time. Scale bar= 20μm. **b.** Quantification shows a decrease in mean fluorescence intensity in both WT and KO neurons over time. This is likely due to bleaching of the background fluorescence suggesting that 568-PFF uptake had not occurred under these conditions. Of note, asterisk shows that PFF are recruited to the membranes along the whole neuron (dendrites, spines and cell body). This was observed on similar time scales between WT and KO neurons (data not shown).

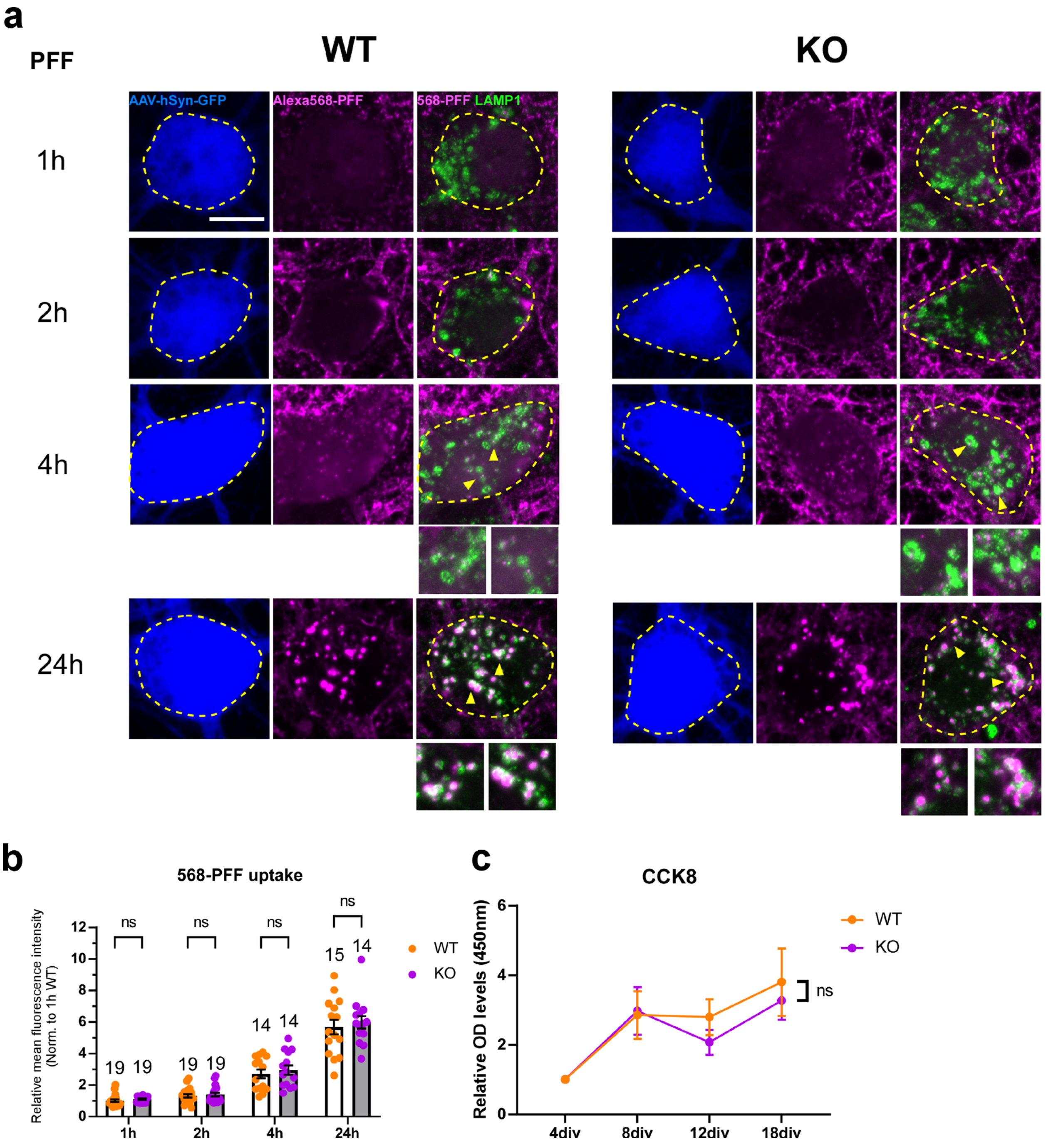

Supplementary Fig. 5: PFF uptake is indifferent between WT and KO neurons.

a. Micrographs show a time course experiment of Alexa568-PFF (magenta) uptake in primary WT and KO neurons transduced to express GFP (blue) and immunolabeled for LAMP1 (green, lysosomal marker). Scale bar= 10 µm. **b.** Quantification shows mean red fluorescence intensity measured in the cell body over 24h post-PFF treatment. n= 3 independent cultures. Numbers above bars mark total number of

neurons analyzed (>4 neurons per culture). Data are mean ±s.e.m. Overall, very little uptake, if any, takes place in the first 2h post-PFF treatment. PFF puncta are clearly seen in colocalization with LAMP1 at the 4h time-point (higher magnification insets) as well as an increased diffused red signal in the cell body cytoplasm. At 24h, much of the membrane-recruited PFF (can be seen as strong signal around the cell body at previous timepoints) are now internalized and trafficked to lysosomes. **c.** Cell counting kit (CCK8) was used to assess differences in cell viability between USP19 WT and KO neurons during maturation in culture. Four time points (4, 8, 12 and 18 DiV) were assessed. n=3 independent primary cultures, each with three technical replicates (wells) per timepoint. Data are mean ±s.e.m. Two-way ANOVA followed by Tukey's multiple comparison test was used for statistical analysis.

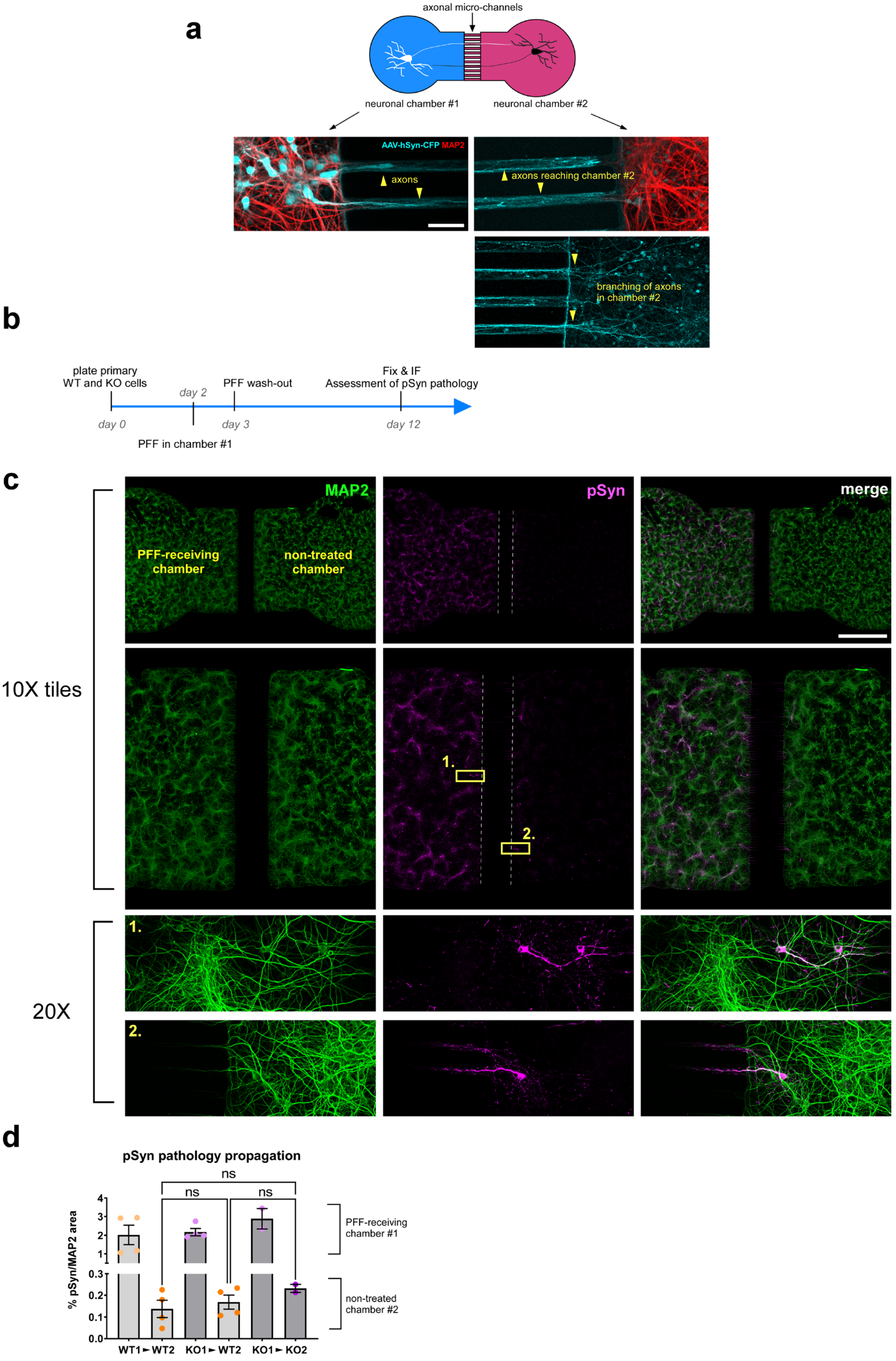

a
axonal micro-channels
neuronal chamber #1
neuronal chamber #2
AAV-hSyn-CFP MAP2
axons
axons reaching chamber #2
branching of axons in chamber #2
b
plate primary WT and KO cells
day 0
day 2
PFF in chamber #1
PFF wash-out
day 3
Fix & IF
Assessment of pSyn pathology
day 12
c
MAP2
pSyn
merge
PFF-receiving chamber
non-treated chamber
10X tiles
20X
1.
2.
d
pSyn pathology propagation
ns
ns
ns
% pSyn/MAP2 area
PFF-receiving chamber #1
non-treated chamber #2
WT1 ► WT2
KO1 ► WT2
KO1 ► KO2

Supplementary Fig. 6: Loss of USP19 does not affect neuron-to-neuron propagation of pSyn pathology

a. Scheme of a microfluidic device (Omega, Enuvio) composed of two neuronal chambers connected by axonal microchannels. Micrographs show good experimental conditions needed to study cell-to-cell propagation where axons of neurons in chamber #1 transduced to express GFP successfully reach to and branch out in chamber #2. Scale bar= 50µm. **b.** Scheme of cell-to-cell propagation experiment. **c.** Micrographs of the cell-to-cell propagation experiment. Whole chamber tile images were acquired at 10X using confocal microscope. Scale bar= 2mm. Insets at 20X show that neurons in both chambers #1 and #2 look healthy and develop $pSyn^{+}$ inclusions. **d.** Quantification of pSyn pathology in PFF-receiving and opposed chambers in WT to WT, WT to KO and KO to KO conditions. n= 2-4 independent cultures. One-way ANOVA was used for statistical analysis followed by Tukey's multiple comparisons test. Data are mean ±s.e.m.

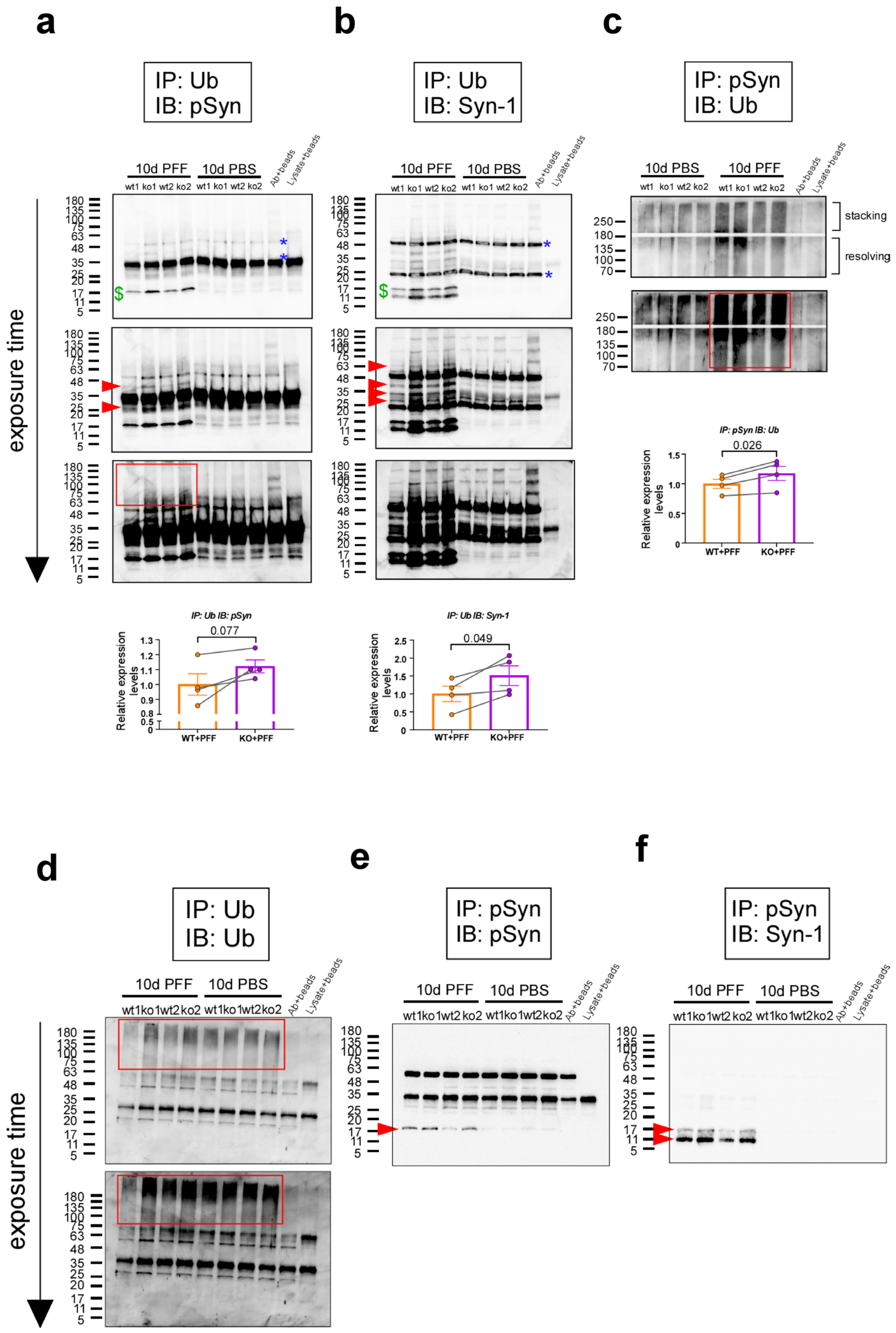
a
IP: Ub
IB: pSyn
10d PFF
10d PBS
wt1 ko1 wt2 ko2 wt1 ko1 wt2 ko2
Ab+beads
Lysate+beads
exposure time
$
*
IP: Ub IB: pSyn
0.077
Relative expression levels
WT+PFF
KO+PFF
b
IP: Ub
IB: Syn-1
10d PFF
10d PBS
wt1 ko1 wt2 ko2 wt1 ko1 wt2 ko2
Ab+beads
Lysate+beads
$
*
IP: Ub IB: Syn-1
0.049
Relative expression levels
WT+PFF
KO+PFF
c
IP: pSyn
IB: Ub
10d PBS
10d PFF
wt1 ko1 wt2 ko2 wt1 ko1 wt2 ko2
Ab+beads
Lysate+beads
stacking
resolving
IP: pSyn IB: Ub
0.026
Relative expression levels
WT+PFF
KO+PFF
d
IP: Ub
IB: Ub
10d PFF
10d PBS
wt1 ko1 wt2 ko2 wt1 ko1 wt2 ko2
Ab+beads
Lysate+beads
exposure time
e
IP: pSyn
IB: pSyn
10d PFF
10d PBS
wt1 ko1 wt2 ko2 wt1 ko1 wt2 ko2
Ab+beads
Lysate+beads
f
IP: pSyn
IB: Syn-1
10d PFF
10d PBS
wt1 ko1 wt2 ko2 wt1 ko1 wt2 ko2
Ab+beads
Lysate+beads

Supplementary Fig. 7: Loss of USP19 enhances ubiquitination of α-syn **in primary cortical neurons.** Immunoprecipitation (non-denaturing conditions) of ubiquitinated proteins from WT and KO neurons at 10dpt using anti-Ub antibody. IP samples (40% of total) were blotted for pSyn (**a**) and total α-syn (Syn-1, **b**). Graphs show whole lane quantification of PFF treated IP samples. Dollar symbols point to monomeric bands at the expected size of monomeric pSyn and α-syn. These mostly likely represent species associated with ubiquitinated forms of pSyn or α-syn under these non-denaturing conditions. Arrowheads points to other specific Ub-pSyn and Ub- α-syn bands. Red rectangle shows high Ub-pSyn M.W. species. Blue asterisks point to heavy and light immunoglobulin chains. **c.** Reverse immunoprecipitation using anti-pSyn antibody. Both stacking and resolving gels were transferred and blotted for the presence of Ub-pSyn. Graph shows the quantification of high M.W. Ub-pSyn of PFF treated samples. n= 4 biologically independent cultures. Paired t-test was used for statistical analysis. Data are means ±s.e.m.. P-values are indicated. **d-f.** Immunoblot images show successful immunoprecipitation of ubiquitinated proteins and pSyn from WT and KO neurons at 10dpt using anti-Ub and anti-pSyn antibodies, respectively. IP samples (10% of total) were blotted for Ub **(d)**, pSyn **(e)** and total α-syn (Syn-1) **(f)**. Rectangles show high M.W. Ub-proteins. Arrowheads point to pulled down pSyn and α-syn.

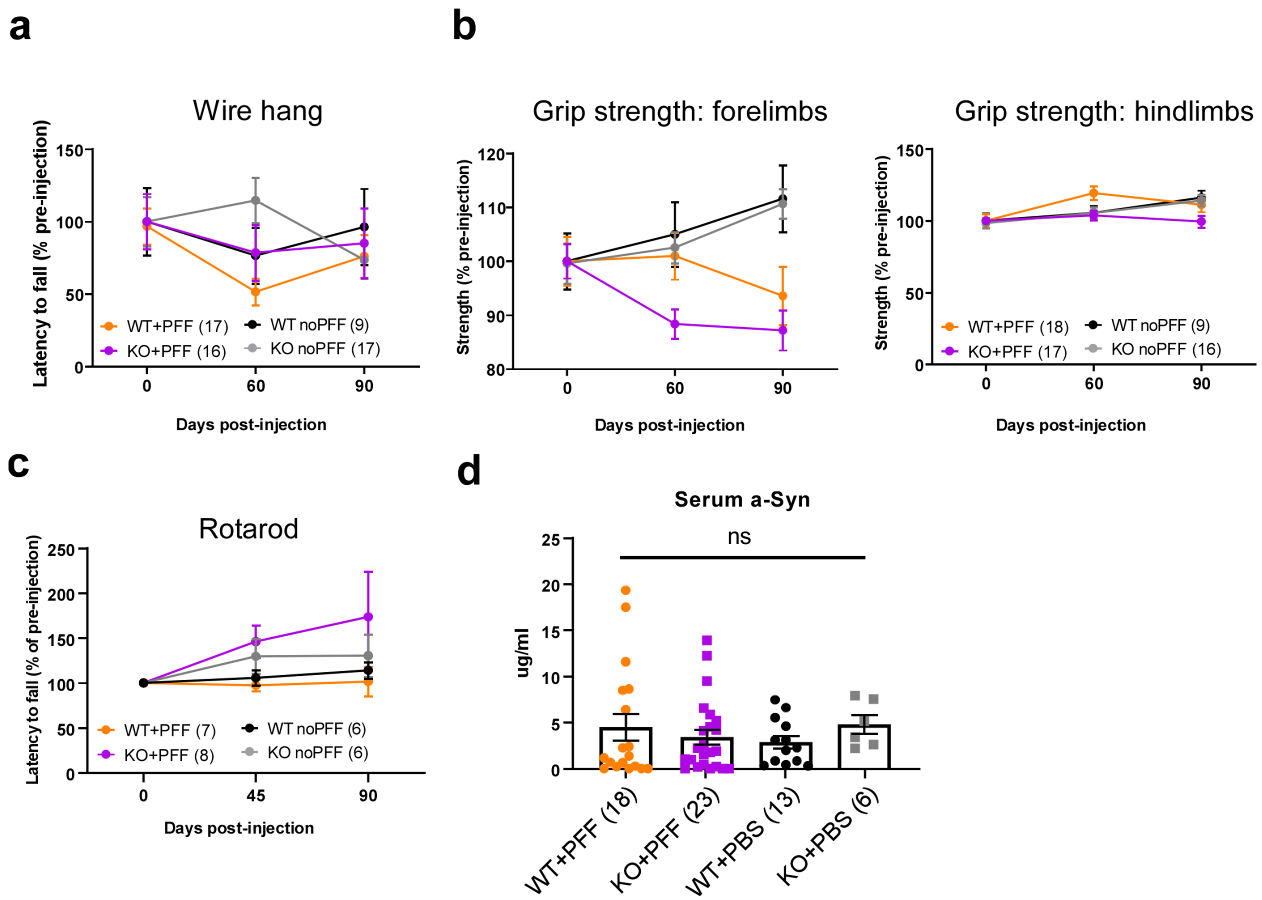

Supplementary Fig. 8: Behavioral analyses and a-syn serum levels in USP19 WT and KO mice. All behavioral tests were performed at three time points: pre-PFF, 1.5-2 and 3-months post-PFF. Non-injected age-matched animals were used as controls. Number of biologically independent animals are indicated in brackets. **a.** Wire-hang (latency to fall). **b.** Grip strength in forelimbs and hindlimbs. **c.** Rotarod (latency to fall). Data are mean ±s.e.m. Two-way ANOVA with Tukey's multiple comparison test was used for statistical analysis. **d.** Graph shows quantification of a-syn levels in serum at end-point (90-120 dpi) and age-matched PBS-injected controls. Data are mean ±s.e.m. One-way ANOVA with Tukey's multiple comparison test was used for statistical analysis.

Supplementary methods

Wire hang

The wire hang test of motor function was conducted using a modified protocol previously described (1). A mouse was placed on the top of a standard wire cage lid. The lid was lightly shaken to cause the mouse to grip to the wires and then turned upside down onto a 40 cm tall empty box. The latency to fall off the wire grid was measured. A trial was stopped if a mouse remained on the lid longer than 15 min. Three different time points (pre-injection, 60 and 90 dpi) were assessed. The animals were not subjected to prior habituation.

Grip strength

Forelimbs and hindlimb strength were measured using a Grip Strength Meter (Columbus Instruments, Columbus, OH, USA). Habituation (4 trials per each leg pair) to the apparatus was performed one day before the test. Average of 4 consecutive trials was used for analysis on the test day. Three different time points (pre-injection, 60 and 90 dpi) were assessed.

Rota-Rod

To assess motor learning, coordination, and balance, mice were subjected to the Rota-Rod test (Stoelting Co., Ugo Basile) as previously done in (1) with minor modifications. Briefly, each mouse was given a training session (three 5-min trials, 5 min apart) to acclimate them to the apparatus. During the test period (2 hr later), each mouse was placed on the rotarod with increasing speed, from 4 rpm to 40 rpm for the period of 5 min. The latency to fall off the rotarod was recorded. Each mouse received two consecutive trials and the mean latency to fall was used for analysis. Three different time points (pre-injection, 45 and 90 dpi) were assessed.

Measurement a-syn in mouse serum

End-point blood collection was performed via cardiac puncture. Blood was let to coagulate for 15 min at room temperature. The clot was removed by centrifugation at 2000x g for 10 min. Serum supernatant was collected and stored at -80°C. Total human aSyn quantification by ELISA was performed using the Human alpha-Synuclein ELISA Kit (Thermo Fisher #KHB0061) following the manufacturer's protocol.

1. Luk KC, Kehm V, Carroll J, Zhang B, O'Brien P, Trojanowski JQ, et al. Pathological alpha-synuclein transmission initiates Parkinson-like neurodegeneration in nontransgenic mice. Science. 2012;338(6109):949-53.

Western Blotting – uncropped blots

Figure 1

c

IB: USP19

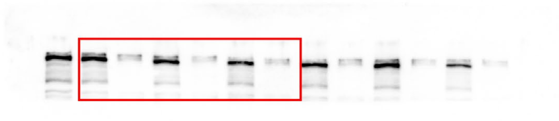

AMIDO BLACK

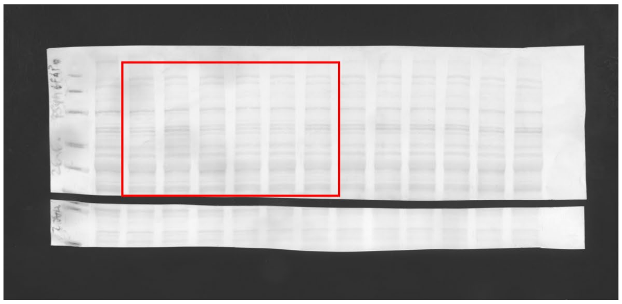

levels adjusted for clearer visualization

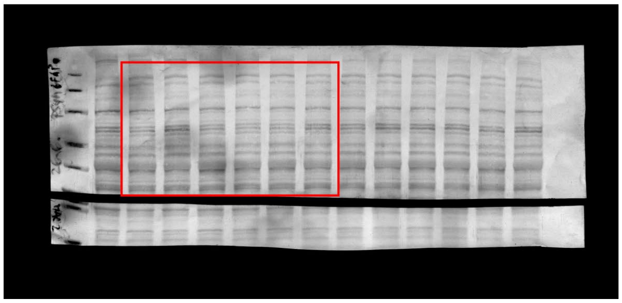

Figure 2

g

IB: pSyn short exp

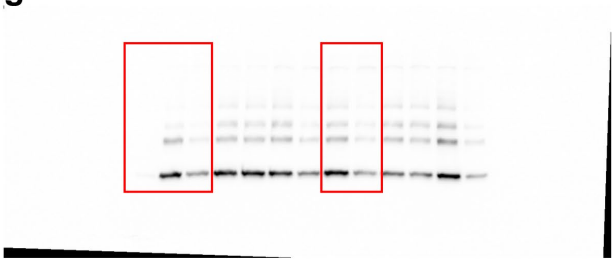

IB: pSyn long exp

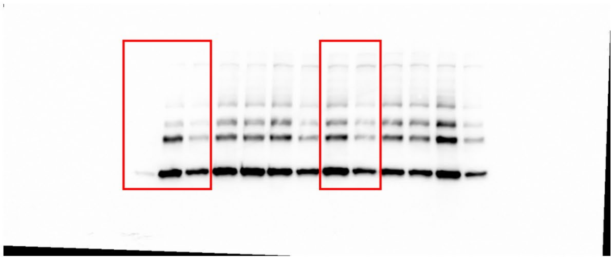

IB: Syn-1 short exp

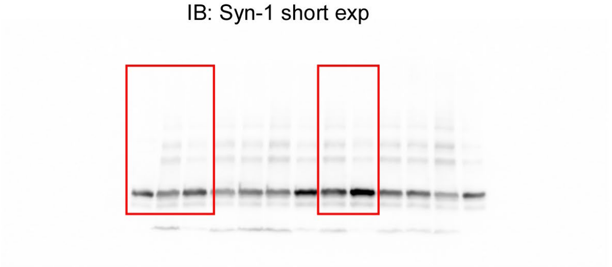

IB:Syn-1 long exp

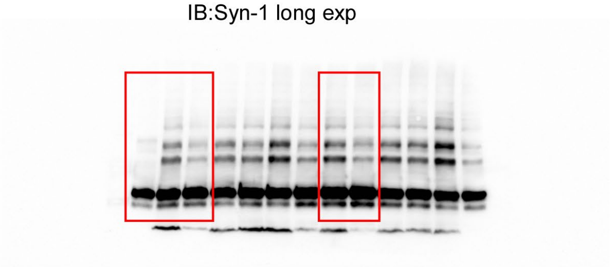

AMIDO BLACK

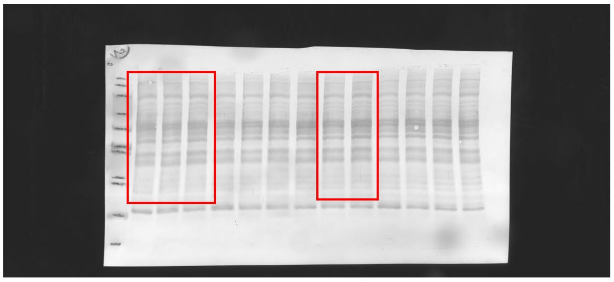

levels adjust for clearer visualization

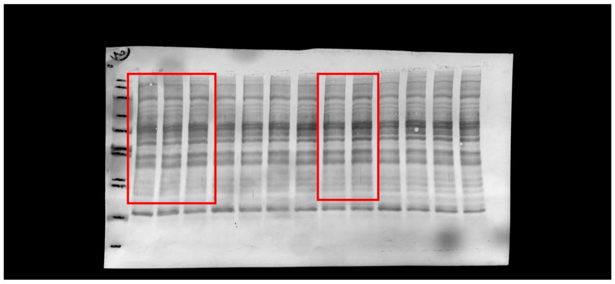

i

IB: pTAU

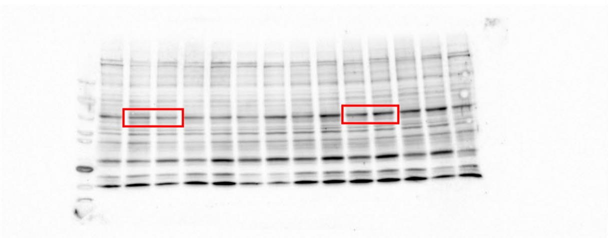

IB: TAU-5

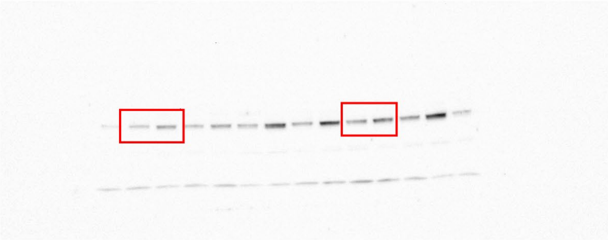

AMIDO BLACK

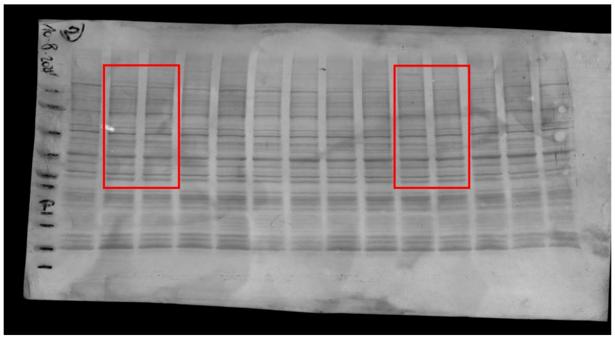

Figure 3

e

IB: GFAP

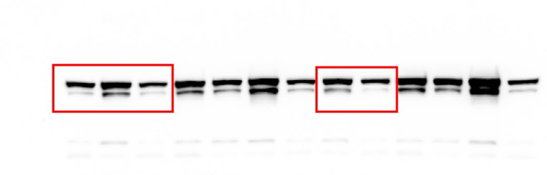

AMIDO BLACK

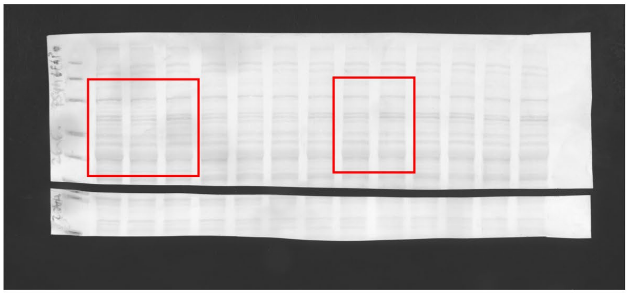

levels adjusted for better visualization

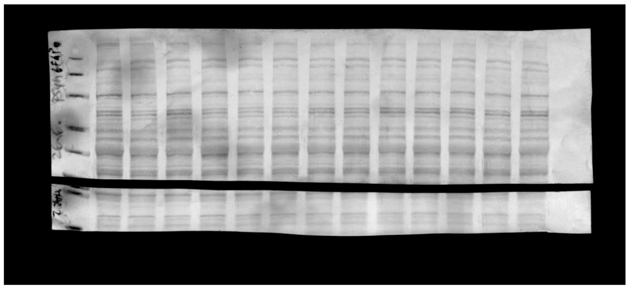

IB: b-actin

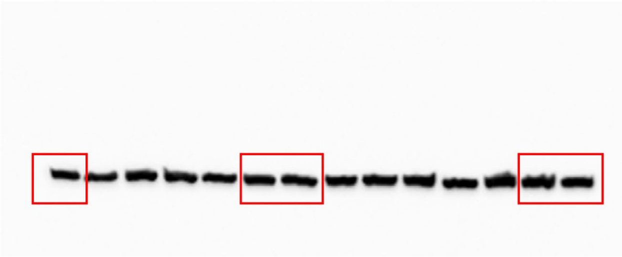

IB: Iba-1

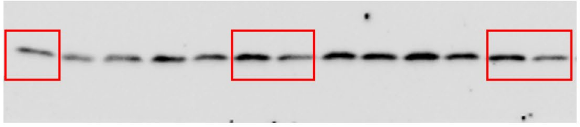

Figure 4

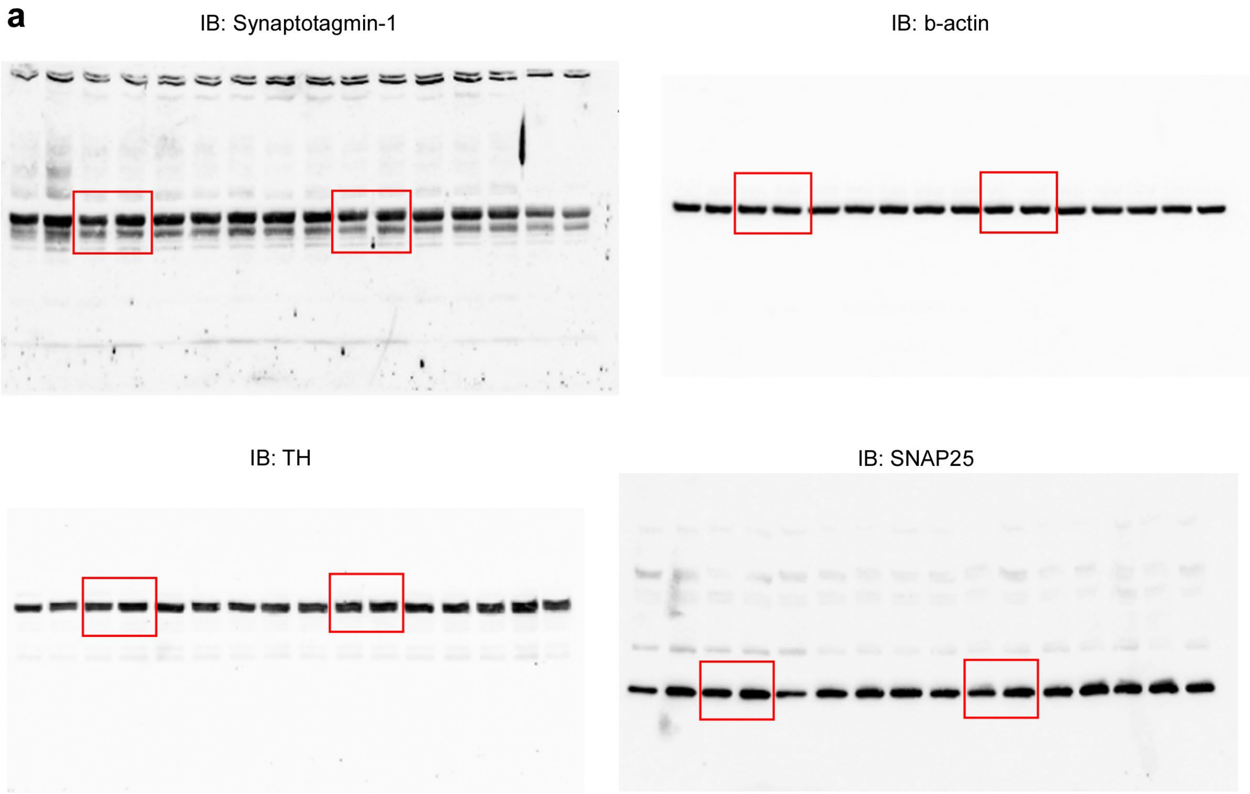

Figure 5

d

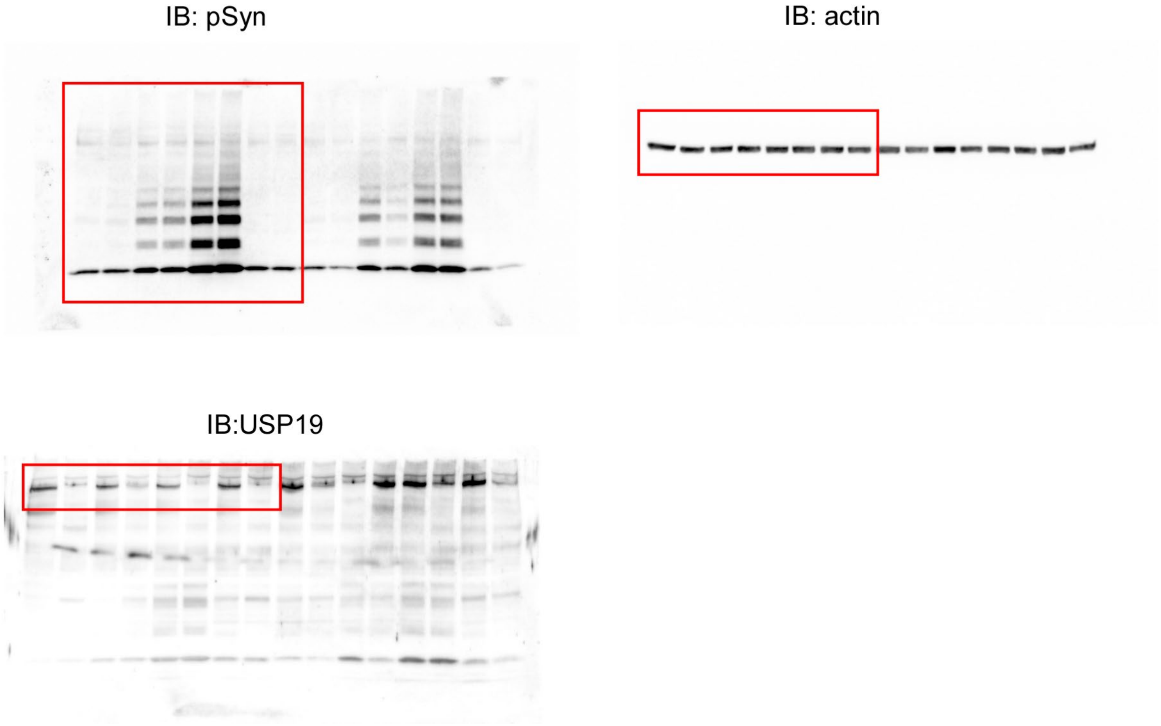

Figure 6

d

IP - pSyn
IB - pSyn short exp

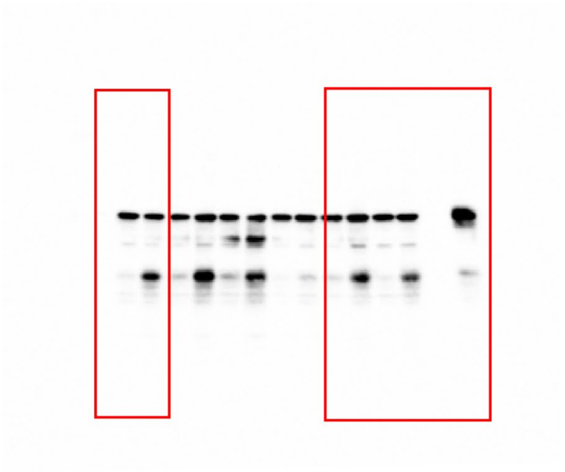

IP - pSyn
IB - pSyn long exp

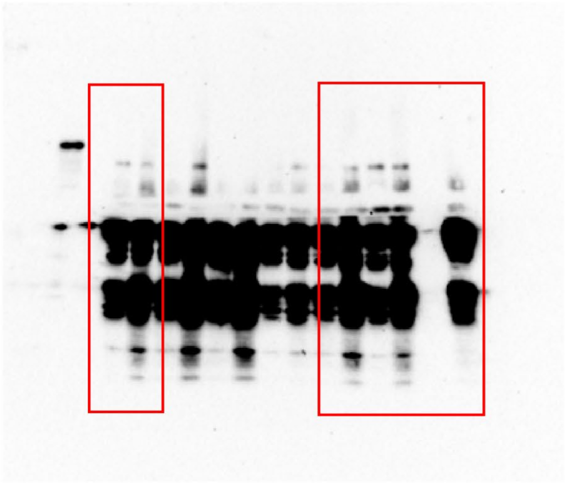

IP - pSyn
IB - Ub

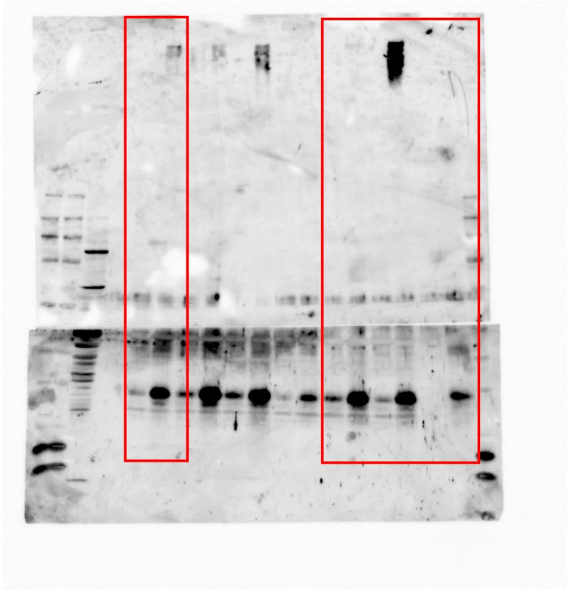

Supplementary figure 2

d, e

IB: pSyn

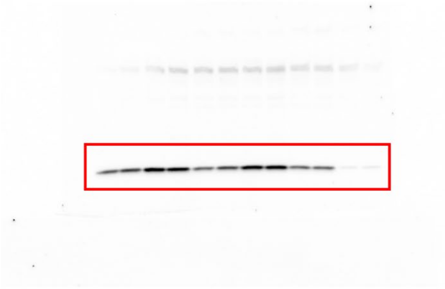

IB: Syn-1

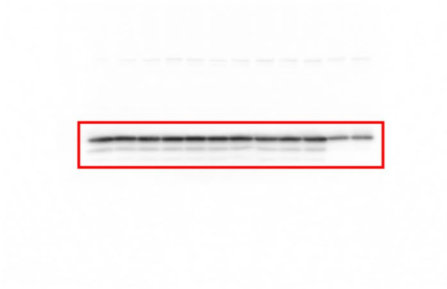

IB: b-actin

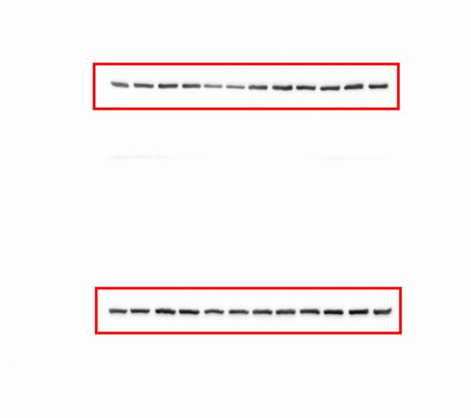

Supplementary figure 7

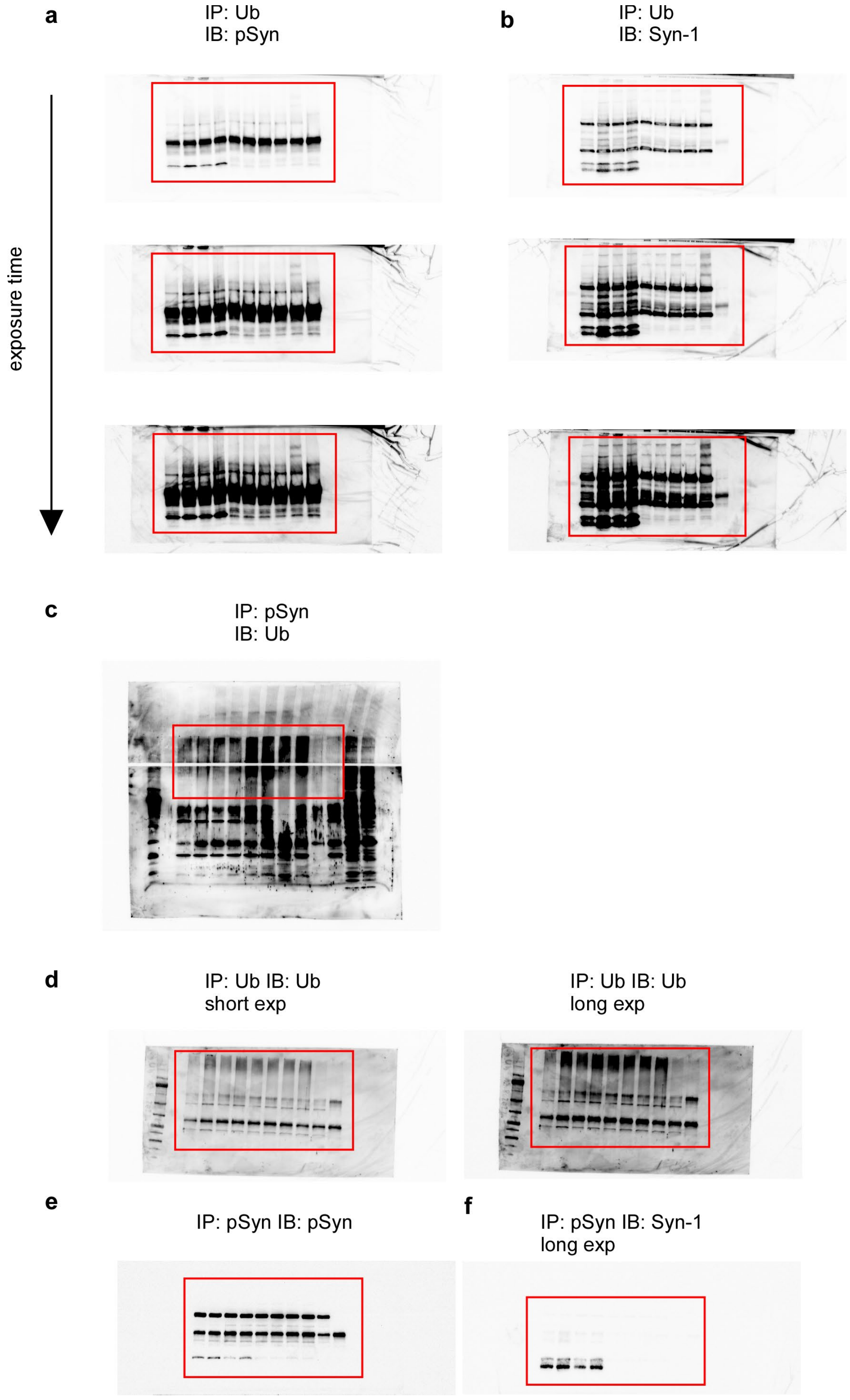

Supplement: Supplementary file 1 — Supplementary material [file 41531_2023_601_MOESM1_ESM.pdf]
