# Supplementary material for: USP19 deubiquitinase inactivation regulates α-synuclein ubiquitination and inhibits accumulation of Lewy body-like aggregates in mice
Source: NPJ Parkinsons Dis. 2023 Nov 28;9:157. doi: 10.1038/s41531-023-00601-1 (PMC10684503; doi:10.1038/s41531-023-00601-1)
Supplement: Supplementary file 2 — Reporting Summary [file 41531_2023_601_MOESM2_ESM.pdf]

nature portfolio

Corresponding author(s): Simon WING and Thomas DURCAN

Last updated by author(s): Nov 7, 2023

Reporting Summary

Nature Portfolio wishes to improve the reproducibility of the work that we publish. This form provides structure for consistency and transparency in reporting. For further information on Nature Portfolio policies, see our Editorial Policies and the Editorial Policy Checklist.

Statistics

For all statistical analyses, confirm that the following items are present in the figure legend, table legend, main text, or Methods section.

n/a	Confirmed	
☐	☒	The exact sample size (n) for each experimental group/condition, given as a discrete number and unit of measurement
☐	☒	A statement on whether measurements were taken from distinct samples or whether the same sample was measured repeatedly
☐	☒	The statistical test(s) used AND whether they are one- or two-sided<br>*Only common tests should be described solely by name; describe more complex techniques in the Methods section.*
☒	☐	A description of all covariates tested
☐	☒	A description of any assumptions or corrections, such as tests of normality and adjustment for multiple comparisons
☐	☒	A full description of the statistical parameters including central tendency (e.g. means) or other basic estimates (e.g. regression coefficient) AND variation (e.g. standard deviation) or associated estimates of uncertainty (e.g. confidence intervals)
☐	☒	For null hypothesis testing, the test statistic (e.g. F, t, r) with confidence intervals, effect sizes, degrees of freedom and P value noted<br>*Give P values as exact values whenever suitable.*
☒	☐	For Bayesian analysis, information on the choice of priors and Markov chain Monte Carlo settings
☒	☐	For hierarchical and complex designs, identification of the appropriate level for tests and full reporting of outcomes
☒	☐	Estimates of effect sizes (e.g. Cohen's d, Pearson's r), indicating how they were calculated

Our web collection on statistics for biologists contains articles on many of the points above.

Software and code

Policy information about availability of computer code

Data collection	Behavioral studies took place in a mouse behavioral testing room using ANY-maze software (Stoelting) in conjunction with a video camera to track and record behavior.
Data analysis	Quantitative image analyses were performed using ImageJ and QuPath software. Behavioral data were analyzed using ANY-maze software (Stoelting). Statistical analysis and graphs were generated with GraphPad Prism 9.

For manuscripts utilizing custom algorithms or software that are central to the research but not yet described in published literature, software must be made available to editors and reviewers. We strongly encourage code deposition in a community repository (e.g. GitHub). See the Nature Portfolio guidelines for submitting code & software for further information.

Data

Policy information about availability of data

All manuscripts must include a data availability statement. This statement should provide the following information, where applicable:

- Accession codes, unique identifiers, or web links for publicly available datasets
- A description of any restrictions on data availability
- For clinical datasets or third party data, please ensure that the statement adheres to our policy

The authors declare that all data supporting the findings of this study are available within the paper and its supplementary information file.

Research involving human participants, their data, or biological material

Policy information about studies with human participants or human data. See also policy information about sex, gender (identity/presentation), and sexual orientation and race, ethnicity and racism.

Reporting on sex and gender	N/A
Reporting on race, ethnicity, or other socially relevant groupings	N/A
Population characteristics	N/A
Recruitment	N/A
Ethics oversight	N/A

Note that full information on the approval of the study protocol must also be provided in the manuscript.

Field-specific reporting

Please select the one below that is the best fit for your research. If you are not sure, read the appropriate sections before making your selection.

☒ Life sciences ☐ Behavioural & social sciences ☐ Ecological, evolutionary & environmental sciences

For a reference copy of the document with all sections, see nature.com/documents/nr-reporting-summary-flat.pdf

Life sciences study design

All studies must disclose on these points even when the disclosure is negative.

Sample size	Sample size calculations were not performed. Numbers of animals and biological replicates were initially based on prior similar studies in our lab or in the literature. We used similar numbers of male and female mice. Studies were repeated (biological replicates) until consistent results were obtained. The latter was assessed by both qualitative direction of each repetition and by statistical analyses of all the data which indicated that the probability of the result occurring by chance was less that 1 in 20 and often less than 1 in 100.
Data exclusions	No data was excluded from any study that was carried out in a technically satisfactory manner.
Replication	As indicated above, studies were replicated until consistent results were obtained.
Randomization	Mice in any given genotype were allocated to treatment groups in a random manner.
Blinding	Investigators were not blinded to group allocation if the downstream measurements were obtained using instruments which would yield measurements in units with absolute values in an operator independent manner. Investigators were blinded to group allocation for behavioral studies where operator involvement could interfere with the results - e.g. grip strength in mice.

Reporting for specific materials, systems and methods

We require information from authors about some types of materials, experimental systems and methods used in many studies. Here, indicate whether each material, system or method listed is relevant to your study. If you are not sure if a list item applies to your research, read the appropriate section before selecting a response.

Materials & experimental systems

n/a	Involved in the study	
☐	☒	Antibodies
☒	☐	Eukaryotic cell lines
☒	☐	Palaeontology and archaeology
☐	☒	Animals and other organisms
☒	☐	Clinical data
☒	☐	Dual use research of concern
☒	☐	Plants

Methods

n/a	Involved in the study	
☒	☐	ChIP-seq
☒	☐	Flow cytometry
☒	☐	MRI-based neuroimaging

Antibodies

Antibodies used	Mouse anti-a-Synuclein (Syn-1) BD Bioscience 610787<br>Mouse anti-alpha-synuclein (4D6) Abcam ab1903<br>Mouse anti-ubiquitin (FK2) Millipore ST1200<br>Rabbit anti-ubiquitin Sigma U5379<br>Rabbit anti-pS129-a-Synuclein (EP1536Y) Abcam ab51253<br>Mouse anti-pS129-a-Synuclein (81A) Abcam ab184674<br>Rabbit anti-phospho-a-Synuclein (Ser129) (D1R1R) Cell Signaling 23706<br>Guinea pig anti-MAP2 Synaptic Systems 188004<br>Rabbit anti-Tyrosine Hydroxylase (TH) Millipore AB152<br>Rabbit anti-NeuN Abcam ab177487<br>Rabbit anti-NeuN Millipore MAB377<br>Mouse anti-Iba-1 Wako 019-19741<br>Rabbit anti-Iba-1 Wako 016-20001<br>Rabbit anti-GFAP Abcam ab7260<br>Mouse anti-GFAP Santa Cruz sc-33673<br>Mouse anti-GFAP Millipore MAB360<br>Mouse anti-pTau (Ser202, Thr205) AT8 Thermo Scientific MN1020<br>Mouse anti-Tau (TAU-5) Thermo Scientific AHB0042<br>Rabbit anti-USP19 Abcam ab167059<br>Mouse anti-SNAP25 Synaptic Systems 111011<br>Mouse anti-Synaptotagmin-1 Synaptic Systems 105011
Validation	All antibodies are from commercial sources with ample data describing their specificity on the manufacturers website, in addition to validation data in our manuscript.

Animals and other research organisms

Policy information about studies involving animals; ARRIVE guidelines recommended for reporting animal research, and Sex and Gender in Research

Laboratory animals	M83 hemizygous (M83hem) mice expressing human alpha-synuclein with the A53T mutation under the control of a prion promoter (Pmp-SNCA*A53T;40) were obtained from The Jackson Laboratory. M83+/+ mice were mated with Usp19−/−/C57BL/6 mice (generated in our laboratory as previously described) for two generations to obtain M83+/+/USP19+/-. As M83hem mice were used in this study, M83+/+/USP19+/- mice were bred with USP19+/- negative for M83 transgene to finally generate USP19+/+/M83hem (USP19 WT/M83hem) and USP19-/-/M83hem (USP19 KO/M83hem).
Wild animals	N/A
Reporting on sex	We studied both female and male mice for each genotype and generally presented the data in an aggregate manner. Sex specific analyses were also carried out (Supplementary figures 2F, 2G, 3A) and indicated interestingly that the effects of USP19 KO were more apparent in females.
Field-collected samples	N/A
Ethics oversight	All animal studies were conducted in accordance with guidelines of the Canadian Council on Animal Care and approved by the Animal Care Committee of the Research Institute of the McGill University Health Centre.

Note that full information on the approval of the study protocol must also be provided in the manuscript.

Plants

Seed stocks	N/A
Novel plant genotypes	N/A
Authentication	N/A
